# Hypophosphatemia during continuous veno-venous hemofiltration is associated with mortality in critically ill patients with acute kidney injury

**DOI:** 10.1186/cc12900

**Published:** 2013-09-19

**Authors:** Yi Yang, Ping Zhang, Yu Cui, Xia Bing Lang, Jing Yuan, Hua Jiang, Wen Hua Lei, Rong Lv, Yi Ling Zhu, En Yin Lai, Jiang Hua Chen

**Affiliations:** 1 Kidney Disease Center, The First Affiliated Hospital, College of Medicine, Zhejiang University, 79 Qingchun Road, Hangzhou 310003, China; 2 Department of Physiology, Zhejiang University School of Medicine, Hangzhou China; 3 Hypertension, Kidney and Vascular Research Center, Georgetown University, Washington, DC USA

## Abstract

**Introduction:**

The primary aim of this study was to determine whether hypophosphatemia during continuous veno-venous hemofiltration (CVVH) is associated with the global outcome of critically ill patients with acute kidney injury (AKI).

**Methods:**

760 patients diagnosed with AKI and had received CVVH therapy were retrospectively recruited. Death during the 28-day period and survival at 28 days after initiation of CVVH were used as endpoints. Demographic and clinical data including serum phosphorus levels were recorded along with clinical outcome. Hypophosphatemia was defined according to the colorimetric method as serum phosphorus levels < 0.81 mmol/L (2.5 mg/dL), and severe hypophosphatemia was defined as serum phosphorus levels < 0.32 mmol/L (1 mg/dL). The ratio of CVVH therapy days with hypophosphatemia over total CVVH therapy days was calculated to reflect the persistence of hypophosphatemia.

**Results:**

The Cox proportional hazard survival model analysis indicated that the incidence of hypophosphatemia or even severe hypophosphatemia was not associated with 28-day mortality independently (*p* = 0.700). Further analysis with the sub-cohort of patients who had developed hypophosphatemia during the CVVH therapy period indicated that the mean ratio of CVVH therapy days with hypophosphatemia over total CVVH therapy days was 0.58, and the ratio independently associated with the global outcome. Compared with the patients with low ratio (< 0.58), those with high ratio (≥ 0.58) conferred a 1.451-fold increase in 28-day mortality rate (95% CI 1.103–1.910, *p* = 0.008).

**Conclusions:**

Hypophosphatemia during CVVH associated with the global clinical outcome of critically ill patients with AKI. The ratio of CVVH therapy days with hypophosphatemia over total CVVH therapy days was independently associated with the 28-day mortality, and high ratio conferred higher mortality rate.

## Introduction

Hypophosphatemia is one of the frequently encountered electrolyte disorders in critically ill patients, with a prevalence ranging from 20% to 40% [1-4] and even reaching 80% in septic patients [5]. Because the common mechanism in hypophosphatemia-caused complications is impaired energy metabolism, hypophosphatemia has also been described as a metabolic disturbance leading to cellular dysfunction in multiple organ systems [6]. Therefore, hypophosphatemia may lead to some serious clinical problems, such as respiratory muscle dysfunction [7,8], decreased myocardial contractility [9], ventricular tachycardia [10] and neuromuscular disturbances [11,12]. Many studies have shown an association between hypophosphatemia and increased mortality [13-15]. However, it remains unclear whether hypophosphatemia actually contributes to mortality or is merely a marker for severity of illness.

Three main mechanisms lead to hypophosphatemia: decreased intestinal absorption, increased renal excretion and internal redistribution of inorganic phosphate. The main causes of hypophosphatemia in critically ill patients include severe infection, trauma, postoperative state, malnutrition, respiratory alkalosis and diabetic ketoacidosis [6]. Renal replacement therapy may also lead to hypophosphatemia, particularly for a prolonged period [16-18]. The incidence of hypophosphatemia was reported to range from 54.0% to 65.1% in the Randomized Evaluation of Normal vs. Augmented Level (RENAL) Replacement Therapy Trial and from 10.9% to 17.6% in the VA/NIH Acute Renal Failure Trial Network (ATN) Study [19,20]. Because renal replacement therapy is one of the most widely used methods to treat critical illness, especially acute kidney injury (AKI), it is crucial to study the effects of high-incidence complications on survival, including hypophosphatemia. Unfortunately, few epidemiologic data about renal replacement therapy associated hypophosphatemia and global clinical outcome have been reported to date.

In the present study, we retrospectively investigated a critically ill patient cohort with AKI who received continuous veno-venous hemofiltration (CVVH) therapy to determine whether hypophosphatemia during CVVH is associated with mortality.

## Materials and methods

This study was approved by the Institutional Ethics Committee of Zhejiang University, and informed consent was obtained from the patients and/or their guardians.

### Patient selection

Between 1 January 2008 and 30 September 2011, about 6,000 critically ill patients brought to the ICU, emergency ICU or surgical ICU at the First Affiliated Hospital, College of Medicine, Zhejiang University, were retrospectively reviewed. From among those patients, 760 individuals who were diagnosed as having AKI and who were receiving CVVH therapy served as the patient group for the present study. AKI was defined according to the risk, injury, failure, loss, and end-stage kidney disease (RIFLE) consensus criteria [21]. Patients who had underlying phosphorus disturbances stemming from premorbidity were not included in the study. Therefore, patients with hypoparathyroidism, hyperparathyroidism, chronic renal tubular defect, alcoholic ketoacidosis, diabetic ketoacidosis and persistent respiratory alkalosis were excluded from the study.

### Patient data

All patient data were abstracted from medical records as well as from the linked clinical inspection database and blood purification database at the hospital. We collected the following demographic and clinical information of the patients at the initiation of and during CVVH therapy: age, gender, premorbidity, recent surgery, main source of infection, Acute Physiology Chronic Health Evaluation II (APACHE II) score at the initiation of CVVH therapy, length of mechanical ventilation after initiation of CVVH therapy, technical parameters and duration of CVVH, phosphate supplementation and daily serum phosphorus level. Serum phosphorus level tests were performed at least once daily at 6:00 AM. Therefore, the data from the 6:00 AM tests were adopted to stand for the daily serum phosphorus levels. Sodium glycerophosphate injection was applied to the patients with hypophosphatemia according the drug manufacturer’s instructions. The clinical principle of the sodium glycerophosphate injection was to maintain the serum phosphorus level at normal standard. Thereafter phosphate supplementation would be sustained, unless the maximum dose permitted by the drug manufacturer’s instructions was reached or hypophosphatemia was corrected. All patients were treated with CVVH. The Accura Hemofiltration System (Baxter Healthcare, Deerfield, IL, USA) was used to administer CVVH therapy. Polysulfone filters (AV600S Dialyzer; Fresenius Medical Care, Bad Homburg, Germany) were used for all patients, and the filter was changed when the transmembrane pressure (TMP) of the filter was greater than 250 mmHg. Central venous access was used with catheters of 11.5Fr or 13.5Fr × 16 cm, 11.5Fr or 13.5Fr × 19.5 cm (Kendall catheter; Tyco Healthcare Group, Mansfield, MA, USA). Nonphosphate replacement fluid was delivered into the extracorporeal circuit at a predilution/postdilution ratio of 2:1. The prescribed dose of CVVH (mean dose in first 72 hours) was 48.53 ml/kg/h. Anticoagulation was performed according to each patient’s condition with unfractionated heparin, low-molecular-weight heparin or heparin-free anticoagulation.

### Variables

As endpoints, we used death during the 28-day period and survival at 28 days after initiation of CVVH. Hypophosphatemia was defined according to the colorimetric method as serum phosphorus level less than 0.81 mmol/L (2.5 mg/dl), and severe hypophosphatemia was defined as serum phosphorus level less than 0.32 mmol/L (1 mg/dl). The ratio of CVVH therapy days with hypophosphatemia to total CVVH therapy days was calculated to reflect the persistence of hypophosphatemia.

### Statistical analysis

Statistical analysis was performed using SPSS version 11.5 software (SPSS, Inc, Chicago, IL, USA). A *P*-value less than 0.05 was considered statistically significant. A univariate comparison was performed to compare variables between two groups using an unpaired *t*-test for continuous variables and a χ^2^ test or Fisher’s exact test for categorical variables. The Cox proportional hazards survival model was applied to identify the independent contribution of prognostic factors to the prediction of outcome at 28 days after initiation of CVVH therapy. When constructing the multivariate model, univariate factors with *P*-values less than 0.2 were used. The odds ratios with 95% confidence intervals (CIs) were used to estimate the association between the independent variables and the dependent variable.

## Results

**Figure 1:**
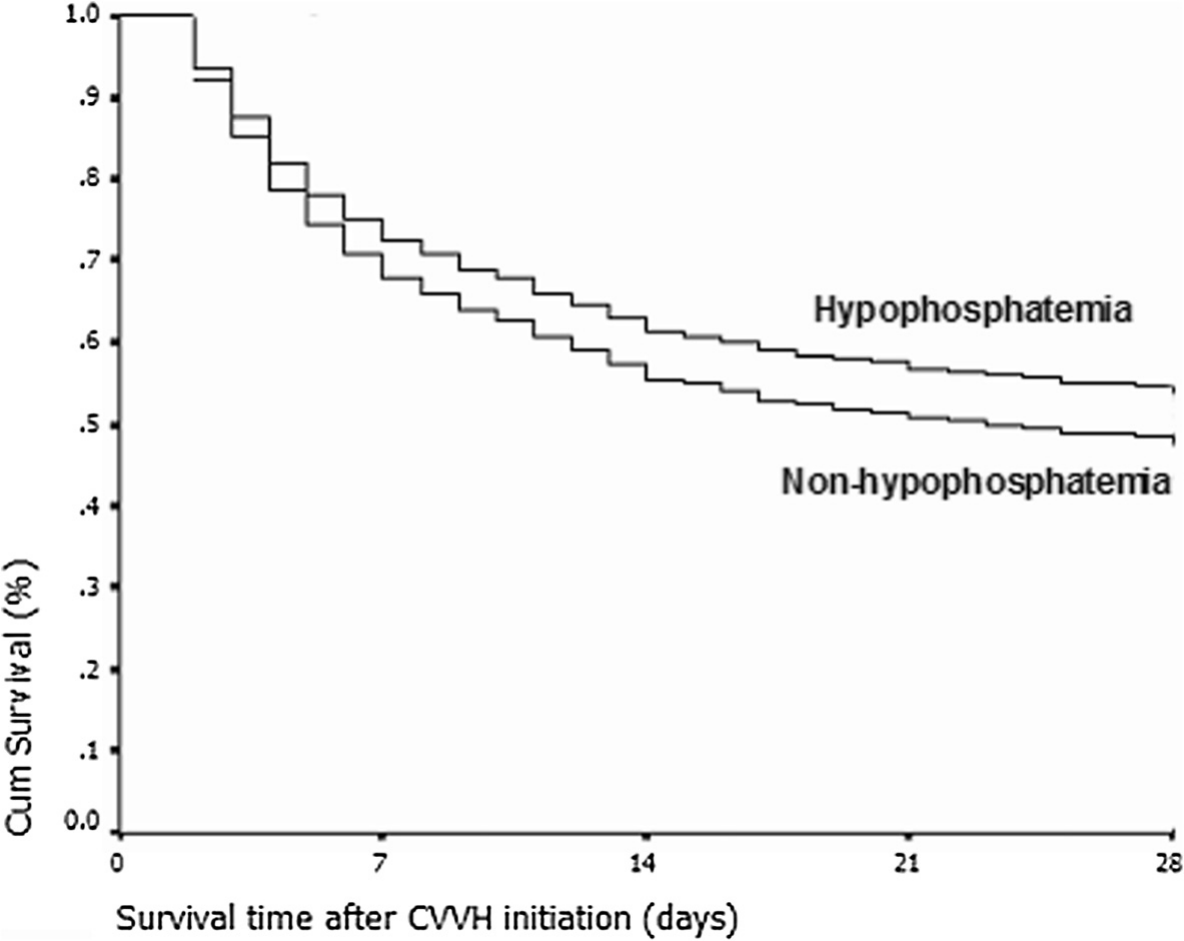
**Cumulative survival rate curve of the Cox proportional hazards survival model.** The curve indicates that the occurrence of severe hypophosphatemia episodes during continuous veno-venous hemofiltration (CVVH) did not significantly increase the 28-day mortality rate of critically ill patients with acute kidney injury compared with those who did not develop severe hypophosphatemia (*P* = 0.700).

The baseline demographics and clinical characteristics of the patients are summarized in Table 1. The mean age (±SD) for patients of the study cohort was 59.72 ± 17.79 years, and 498 patients (65.5%) were male. A total of 406 patients (53.4%) were diagnosed with sepsis according to the criteria published by the International Sepsis Definitions Conference [22]. The mean APACHE II score (±SD) at the initiation of CVVH therapy was 21.04 ± 6.64. A total of 471 patients (62.0%) were injected with sodium glycerophosphate according the manufacturer’s drug instructions. A total of 521 patients (68.6%) had hypophosphatemia episodes during CVVH therapy, and 109 patients (14.3%) developed severe hypophosphatemia according to our definition. A total of 387 patients (50.9%) were alive at day 28 after initiation of CVVH. Univariate analysis indicated that survivors were significantly different from nonsurvivors with regard to some demographic and clinical characteristics, including younger age, less likely to have chronic ill health (as defined by the APACHE II criteria), lower occurrence of sepsis, lower APACHE II scores and lower occurrence of severe hypophosphatemia (11.4% vs. 17.4%; *P* = 0.022). However, gender, recent surgery, median length of mechanical ventilation after initiation of CVVH therapy, median dose and therapeutic time of CVVH, the proportion of patients receiving phosphate supplementation and the occurrence of hypophosphatemia (67.2% vs. 70.0%; *P* = 0.435) demonstrated no significance between the survivor and nonsurvivor cohorts. Univariate factors with *P*-values less than 0.2 included age, chronic ill health, sepsis, APACHE II score and presence or absence of severe hypophosphatemia episode during CVVH therapy. These patients were included in the Cox proportional hazards survival model to avoid the potential influence of some confounding risk factors. The multivariate analysis indicated that the occurrence of severe hypophosphatemia episode during CVVH therapy was dependent on outcome (*P* = 0.700), whereas APACHE II score was independent of 28-day mortality (Figure 1 and Table 2).

**Table 1:** **Baseline demographic and clinical characteristics of the patients**^**a**^

**Characteristics**	**Patients** **(*N *** **= 760)**	**Survivors** **(*n *** **= 387)**	**Nonsurvivors** **(*n *** **= 373)**	***P***
Mean age (yr)	59.72	58.25	61.25	0.020
Male sex (%)	498 (65.5)	250 (64.6)	248 (66.5)	0.594
Premorbidity (%)	496 (65.3)	234 (60.5)	262 (70.2)	0.007
Cardiovascular disease	171	78	93	
Liver disease	94	37	57	
Pulmonary disease	31	15	16	
Cancer	77	34	43	
Renal disease	63	41	22	
Hematological disease	28	10	18	
Other	32	19	13	
Recent surgery (%)	272 (35.8)	138 (35.7)	134 (35.9)	>0.999
Sepsis (%)	406 (53.4)	188 (48.6)	218 (58.4)	0.010
Main source of infection (%)				
Chest	233 (57.3)	97 (51.6)	136 (62.4)	
Abdomen	118 (29.1)	62 (33.0)	56 (25.7)	
Bloodstream, including line-related infection	36 (8.9)	20 (10.6)	16 (7.3)	
Other or unknown source	19 (4.7)	9 (4.8)	10 (4.6)	
APACHE II score (mean)	21.04	19.42	22.72	<0.001
Mean length of mechanical ventilation (days)	8.01	8.30	7.70	0.290
Prescribed dose of CVVH (mean in first 72 hours, ml/kg/h)	48.53	47.67	49.42	0.089
Delivered dose of CVVH (median in first 72 hours, ml/kg/h)	41.00	40.32	41.71	0.542
Therapeutic time of CVVH (days)	7.94	8.20	7.67	0.271
Phosphate supplementation (%)	471 (62.0)	248 (64.1)	223 (59.8)	0.232
Hypophosphatemia during CVVH (%)	521 (68.6)	260 (67.2)	261 (70.0)	0.435
Severe hypophosphatemia (%)	109 (14.3)	44 (11.4)	65 (17.4)	0.022

^a^APACHE II, Acute Physiology Chronic Health Evaluation II; CVVH, continuous veno-venous hemofiltration. *P*-value represents live cohort vs. dead cohort.

**Table 2:** **Variables included in the Cox proportional hazards survival model and hazard ratios**^**a**^

**Characteristics**	**Odd ratio (95% confidence interval)**	***P***
Age	1.001 (0.994 to 1.008)	0.749
Presence of chronic ill health	1.211 (0.941 to 1.558)	0.137
APACHE II score	1.058 (1.041 to 1.076)	<0.001
Presence of sepsis	1.015 (0.806 to 1.279)	0.896
Presence of severe hypophosphatemia episode during CVVH therapy	0.943 (0.699 to 1.272)	0.700

^a^APACHE II, Acute Physiology Chronic Health Evaluation II; CVVH, continuous veno-venous hemofiltration.

**Figure 2:**
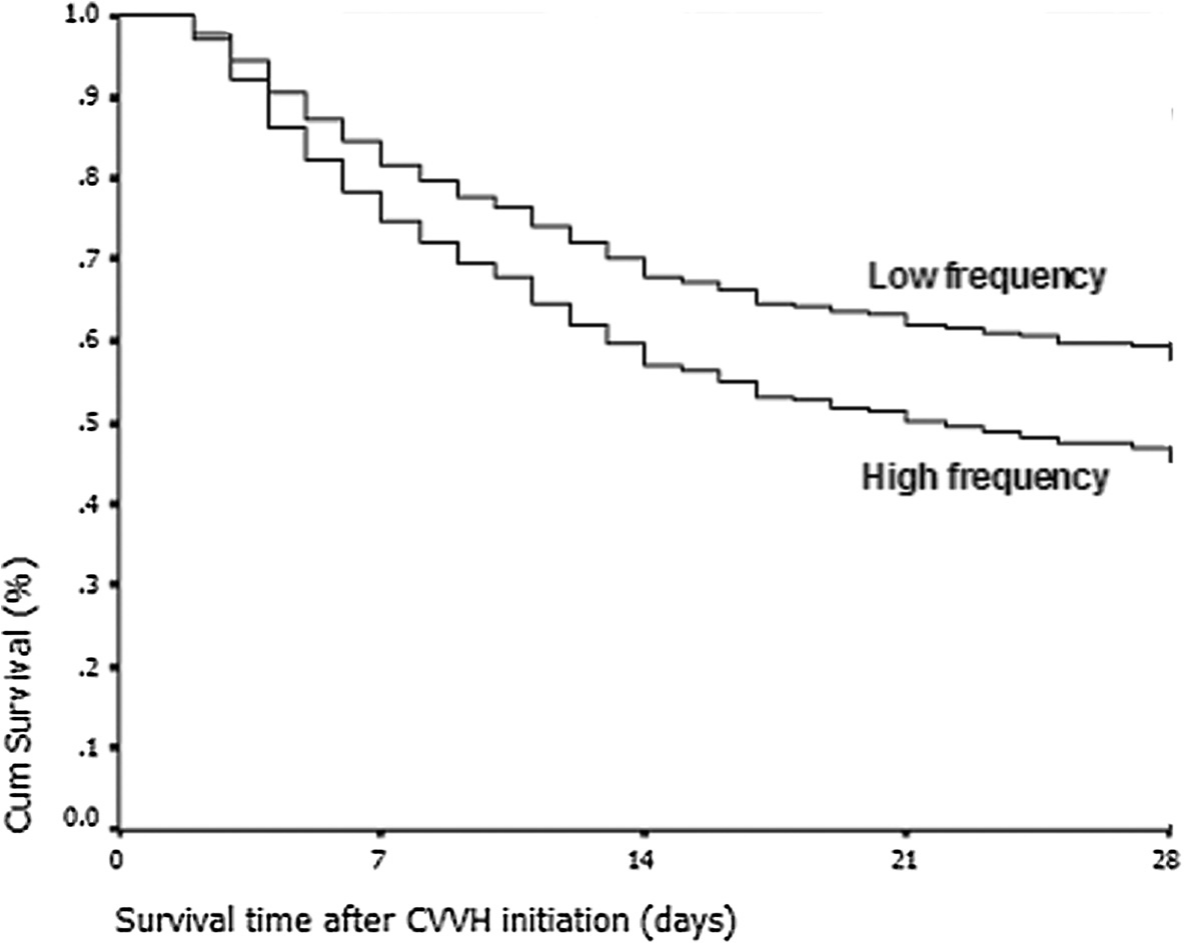
**Cumulative survival rate curve of the Cox proportional hazards survival model.** The survival curve indicates that patients with a high ratio of continuous veno-venous hemofiltration (CVVH)therapy days with hypophosphatemia to total CVVH therapy days (ratio ≥ 0.58) had an increased 28-day mortality rate compared with those with a low ratio (ratio < 0.58) during CVVH therapy (*P* = 0.008).

Thereafter we analyzed the subcohort of 521 patients who had developed hypophosphatemia during the CVVH therapy period. In this cohort, the mean ratio of CVVH therapy days with hypophosphatemia to total CVVH therapy days was 0.58. Therefore, the ratio ≥0.58 was defined as a high ratio, and the ratio <0.58 was defined as a low ratio. A total of 260 patients (49.9%) were alive at day 28 after initiation of CVVH of this cohort, and the univariate analysis indicated that survivors were significantly different from nonsurvivors regarding some factors, including younger age, less likelihood of chronic ill health, a lower occurrence of sepsis, lower APACHE II score and lower occurrence of high ratio of CVVH therapy days with hypophosphatemia to total CVVH therapy days (36.9% vs. 55.9%; *P* < 0.001) (Table 3). However, gender, recent surgery, median length of mechanical ventilation after initiation of CVVH therapy, median dose and therapeutic time of CVVH and proportion of patients receiving phosphate supplementation demonstrated no significance between the survivor and nonsurvivor cohorts (Table 3). Univariate factors with *P*-values less than 0.2, including age, chronic ill health, sepsis, APACHE II score and high or low ratio of CVVH therapy days with hypophosphatemia to total CVVH therapy days were recruited in the Cox proportional hazards survival model and indicated that the ratio of CVVH therapy days with hypophosphatemia to total CVVH therapy days was independently associated with mortality. Compared with the patients with a low ratio, those with a high ratio conferred a 1.451-fold increase in 28-day mortality rate (95% CI = 1.103 to 1.910; *P* = 0.008) (Figure 2 and Table 4). Meanwhile, APACHE II score was also an independent factor associated with 28-day mortality.

**Table 3:** **Baseline demographic and clinical characteristics of the patients who developed hypophosphatemia during the continuous veno-venous hemofiltration therapy period**^**a**^

**Characteristics**	**Patients** **(*N *** **= 521)**	**Survivors** **(*n *** **= 260)**	**Non-survivors** **(*n *** **= 261)**	***P***
Mean age (yr)	60.34	57.97	62.71	0.002
Male sex (%)	326 (62.6)	163 (62.7)	163 (62.5)	>0.999
Premorbidity (%)	347 (66.6)	155 (59.6)	192 (73.6)	0.001
Cardiovascular disease	122	56	66	
Liver disease	66	24	42	
Pulmonary disease	25	12	13	
Cancer	51	21	30	
Renal disease	43	25	18	
Hematological disease	22	7	15	
Other	18	10	8	
Recent surgery (%)	209 (40.1)	104 (40.0)	105 (40.2)	0.929
Sepsis (%)	304 (58.3)	137 (52.7)	167 (64.0)	0.016
Chest	177 (58.2)	68 (49.6)	109 (65.3)	
Abdomen	92 (30.3)	51 (37.2)	41 (24.6)	
Bloodstream, including line-related infection	25 (8.2)	13 (9.5)	12 (7.2)	
Other or unknown source	10 (3.3)	5 (3.6)	5 (3.0)	
APACHE II score (mean)	20.86	19.32	22.44	<0.001
Length of mechanical ventilation (days)	9.56	9.68	9.44	0.700
Prescribed dose of CVVH (mean in first 72 hours, ml/kg/h)	50.78	50.12	51.43	0.272
Delivered dose of CVVH (median in first 72 hours, ml/kg/h)	42.23	41.78	42.68	0.235
Therapeutic time of CVVH (days)	9.49	9.60	9.37	0.710
Phosphate supplementation (%)	355 (68.1)	175 (67.3)	180 (69.0)	0.707
Ratio of CVVH therapy days with hypophosphatemia to total CVVH therapy days (mean)	0.58	0.50	0.65	<0.001
High ratio^b^ (%)	242 (46.4)	96 (36.9)	146 (55.9)	
Low ratio^c^ (%)	279 (53.6)	164 (63.1)	115 (44.1)	

^a^ Acute Physiology Chronic Health Evaluation II; CVVH, continuous veno-venous hemofiltration. *P* value represents live cohort vs. dead cohort. ^b^High ratio means the ratio of CVVH therapy days with hypophosphatemia to total CVVH therapy days ≥0.58. ^c^Low ratio means the ratio of CVVH therapy days with hypophosphatemia to total CVVH therapy days <0.58.

**Table 4:** **Variables included in the Cox proportional hazards survival model and hazard ratios of patients who developed hypophosphatemia during the continuous veno-venous hemofiltration therapy period**^**a**^

**Characteristics**	**Odd ratio (95% confidence interval)**	***P***
Age	1.004 (0.995 to 1.013)	0.373
Presence of chronic ill health	1.284 (0.946 to 1.742)	0.109
APACHE II score	1.051 (1.029 to 1.073)	<0.001
Presence of sepsis	1.049 (0.788 to 1.396)	0.742
Presence of high ratio^b^	1.451 (1.103 to 1.910)	0.008

^a^Acute Physiology Chronic Health Evaluation II. ^b^A high ratio means the ratio of continuous veno-venous hemofiltration (CVVH) therapy days with hypophosphatemia to total CVVH therapy days ≥0.58.

## Discussion

AKI is a frequent problem in the ICU associated with substantial morbidity, mortality and healthcare expenditures. Severe AKI is always accompanied by dysfunction in other organs and is characterized by high mortality. Renal replacement therapy remains the cornerstone of supportive care [23]. To date, some important issues regarding renal replacement therapy are still unclear, and any progress regarding interventions may contribute much to the global outcomes of these critically ill patients. Therefore, in our present study, patients diagnosed with AKI receiving CVVH therapy were recruited as the study cohort to determine the relationship of clinical outcomes and hypophosphatemia, one of the frequently complications of renal replacement therapy.

It has been indicated that, besides CVVH therapy, the main causes of hypophosphatemia in critically ill patients may include severe infection, trauma, postoperative state and malnutrition [6]. Indeed, in the present study, 272 patients (35.8%) had received surgical interventions and 406 patients (53.4%) were diagnosed with sepsis. Therefore, hypophosphatemia may occur in some patients before the initiation of CVVH therapy because of the illness itself. Importantly, the potential that hypophosphatemia could be a surrogate marker for something else can never be excluded, even during the CVVH therapy period.

It was reported in the ATN Study that the incidence of hypophosphatemia was 10.9% in the low-intensity group and 17.6% in the high-intensity group [20]. In the RENAL Trial, the incidence of hypophosphatemia in the low- and high-intensity groups was 54.0% and 65.1%, respectively [19]. The difference in hypophosphatemia incidence may due to the following causes. The modality of renal replacement therapy performed in the ATN Study included intermittent hemodialysis, sustained low-efficiency dialysis and continuous veno-venous hemodiafiltration. In the RENAL Study only continuous veno-venous hemodiafiltration was administered. The therapeutic dose of renal replacement therapy adopted in the ATN Study was lower than that in the RENAL Trial (35 ml/kg/h vs. 40 ml/kg/h in the high-intensity group). The results of comparison of the two study implied that phosphate clearance on continuous veno-venous hemodiafiltration might be significantly greater than intermittent hemodialysis because of ongoing intercompartmental mass transfer and larger filter pore size. In the present study, the modality of renal replacement therapy was CVVH, the mean therapeutic dosage was closer to that used in the RENAL Trial design, and the incidence of CVVH-associated hypophosphatemia according to our data was also similar to the RENAL Trial.

Hypophosphatemia can be caused by decreasing absorption, increasing renal excretion or internal redistribution of inorganic phosphate. In critically ill patients, inorganic phosphate redistribution across the cell membrane is a common phenomenon [24]. Therefore, serum phosphorus levels do not accurately reflect total body phosphorus stores, and this bias can be aggravated by CVVH therapy because of the quick serum phosphorus clearance during the intervention. The ratio of CVVH therapy days with hypophosphatemia to total CVVH therapy days was calculated in the present study to avoid the bias of the serum phosphorus test. The present study showed that a single test of serum phosphorus concentration could not serve as an independent factor predictive of mortality even if it reached the standard of severe hypophosphatemia. Interestingly, the ratio of CVVH therapy days with hypophosphatemia to total CVVH therapy days was proved to be a factor independent of the global clinical outcome through the multivariate analysis, indicating that a high ratio conferred a higher 28-day mortality rate. The results suggested that the persistence of hypophosphatemia had the potential to harm critically ill patients. Once hypophosphatemia happens, the therapeutic strategy might be adjusted, including more phosphate supplementation as well as regulation of renal replacement dosage or modality, to avoid persistence of hypophosphatemia.

To date, whether the impact of intensity or dosage of renal replacement therapy is crucial to the outcomes of critically ill patients is controversial. Despite the benefits of high-intensity renal replacement therapy, including superior hemodynamic stability, metabolic clearance and volume control, no survival advantage can be demonstrated with its use [25]. Although study design may explain much of this paradox, it is also quite plausible that the complications of the high-intensity renal replacement therapy offset its potential benefits in critically ill patients. Unfortunately, some outcome–dose studies have not even discussed adverse effects. In our data, normal phosphate supplementation treatment failed to reverse hypophosphatemia during CVVH because the majority of the patients with hypophosphatemia (68.6%) received regular sodium glycerophosphate injections. Thus, a new strategy should be adopted to resolve this clinical problem. The present study highlights the need for analysis of adverse effects and clinical strategies to correct hypophosphatemia during CVVH therapy, including phosphate supplementation, especially the use of phosphate-containing replacement fluid to decrease the high mortality rate of severe AKI patients [17,18,26].

The present study has several limitations. The retrospective study design decreased the power of the conclusions. As the endpoints we adopted, we dealt with the 28-day mortality in the present study, which was only a surrogate outcome. In addition, various clinical settings may lead to hypophosphatemia in critically ill patients, so we could never exclude the potential that hypophosphatemia could be a surrogate marker for something other than CVVH. A prospective study with various days to mortality may resolve this bias.

## Conclusions

We retrospectively investigated a critically ill patient cohort with AKI receiving CVVH therapy and found that hypophosphatemia during CVVH was associated with 28-day mortality. The ratio of CVVH therapy days with hypophosphatemia to total CVVH therapy days was independently associated with the global outcome. Compared with the patients with low ratios, those with high ratios had a 1.451-fold increase in 28-day mortality rate (95% CI = 1.103 to 1.910; *P* = 0.008). This conclusion implies that the persistence of hypophosphatemia risks potential harm to critically ill patients. Once hypophosphatemia happened, the therapeutic strategy might be adjusted to avoid its persistence.

## Key messages

● Hypophosphatemia during CVVH was associated with the global clinical outcome of critically ill patients with AKI.

● The ratio of CVVH therapy days with hypophosphatemia to total CVVH therapy days was independently associated with 28-day mortality.

● A high ratio of CVVH therapy days with hypophosphatemia to total CVVH therapy days conferred a higher mortality rate among critically ill patients with AKI.

## Abbreviations

AKI: Acute kidney injury; APACHE II: Acute Physiology and Chronic Health Evaluation II; CVVH: Continuous veno-venous hemofiltration; TMP: Transmembrane pressure.

## Competing interests

The authors declare that they have no competing interests.

## Authors’ contributions

YY and JC conceived the study, participated in its design and coordination and drafted the manuscript. PZ participated in the study design and coordination and helped to draft the manuscript. YC and HJ participated in the study design and performed the statistical analysis. XL participated in the experimental activity and contributed to drafting the manuscript. JY participated in the experimental activity and interpretation of the results. WL participated in the experimental activity. RL performed the statistical analysis. YZ participated in the experimental activity. EL discussed the study and revised the manuscript. All authors read and approved the final manuscript.
